# The Reason of the Faith That Is in Me

**Published:** 1897-06

**Authors:** Linaeus C. Shecut

**Affiliations:** Marion, S. C., Member of the Junior Class of the Univ. of Md. Dental Department.


AMERICAN JOURNAL

—OF—

DENTAL SCIENCE.

VOL. XXXI. BALTIMORE, JUNE, 1897. NO. 2.

ARTICLE I.

### THE REASON OF THE FAITH THAT IS IN ME.

BY LINAEUS C. SHECUT, OF MARION, S. C., MEMBER OF THE JUNIOR CLASS OF THE UNIV. OF MD. DENTAL DEPARTMENT.

How often do we hear students asked the question, Why do you select dentistry? And how seldom is it seriously considered! Do we stop to think that the answer conscientiously given is the index to our future? No doubt every case has its own circumstances that lead up to the study of dentistry, but still it should possess an over-ruling purpose that controls these. So, thinking upon the possibilities of our profession it might not be amiss to consider and even dwell upon the spirit that characterizes the young men that are becoming dental students. True it is, some may enter "for money's sake;" but watch the future of such, and though time may look kindly upon them even to the gratification of their desire, though many rise to praise their skill and learning, still the fact that a sordid ambition was the incentive to this end would cause even their masterpieces to hide themselve in reproachful shame at the gaze of honest men. Happily for us, there are not many such. The vast majority possess the true sentiment, and oh! how much more comforting to consider the future of one of these. Though the crown of wealth may not rest upon his brow, though the artistic touch of others may surpass his handiwork; still, the knowledge of an honest purpose nobly pursued will cause the people to praise his goodness, and the profession to honor his record.

So in the beginning, as young men entering upon the field of dentistry, we should fully realize its present scope and future possibilities as a science, then satisfy ourselves that our purpose is to work for its elevation with our best talent and then be true to our purpose, and success will surely crown our labors.

It is a source of great gratification to look out upon the colleges of the country and note the fine quality of intellect and character that stamps the students. It is in these that we must place our trust and stake our hope for the future, for the demands of competency can only be met by such. Our colleges, boards and the profession should mutually work to keep the standard high and its attainment deserved, so that our profession will be made up of men all possessing the element of success. Our age, I fear, is learning to hold sentiment too lightly, caring too little for the spirit of things, being satisfied merely with their accomplishment. We should stop and consider how such things count. Notice it among the men of our profession. See the test of the student at college in the "failure of first effort." As he looks out and contemplates the field of study and labor that lies before him, and when that characteristic feeling that he should know it all at once comes over him, how strongly do the words of Longfellow come back, "Art is long and time is fleeting."

Right here we find the test of the "make-up" of a man. Lucky for the world that he does not remain long in this mood. The other side of the canvas now dawns upon him. He sees brightness ahead, and will sketch you the future with brighter crayon and gild it with golden rays. In a nature of this kind ambitious desire is at its height and needs only time to be satisfied. The bag of gold may be at the rain-bow's end but still he believes he can find it. The twin sisters fame and renown may be cradled far in the bosom of the heavens, nevertheless they are shining there for him. In other words he sees success ahead, and "hope springs eternal in his breast." When we can look out and see our young men with such a spirit as this, we can at once give the reasons of the faith that is in us for the future of dentistry. For what relations do the individual spirits of any profession bear to its advancement? Could dentistry occupy its present place among the sciences if its progenitors had been men of weak minds and irresolute. Who of you that have succeeded, in your youth, did not see over the confronting obstacles the bright future? Can that spirit that sees nothing but darkness ahead and quakes at the first touch ever rise? Never! The spirit that succeeds is the one that brooks no defeat, to whom present difficulties predict a brighter future and present failure is but an assurance of coming success. These are the men we want in our profession, men that have intellect, character and noble ambitions, men given to exploration and research, and above all others Christian men. To obtain such our colleges must do the sifting. Let the standard be in the reach of every honest striver, but let only the fit survive. I believe the majority of the colleges are doing this. It was my pleasure to attend the past course of lectures at the University of Maryland. At this school the students of the three departments, (law, medicine and dentistry), formed a very close relationship, especially was this so between those of medicine and dentistry. Being thrown together in and out of the lecture halls each was constantly learning of the other. They learned not only how closely were they related, but also how clearly were the separating lines of the two professions defined, and as these men mingled together how gratifying was it to see the dental men—from strength of mind, force of character and power of application—stand the peers of any of the rest.

I feel that it is this way in the other colleges. And now as a student, one who has yet the requirements to meet but one who feels that the cause is worthy the best efforts of every seeker, I would say to the colleges—keep on in your course, reaching out for the time when our hopes shall be realized and stop not—

Till teeth shall have ceased to decay
And man has driven all pain away.

---

ARTICLE II.

